# Outcomes of Extensive Hybridization and Introgression in *Epidendrum* (Orchidaceae): Can We Rely on Species Boundaries?

**DOI:** 10.1371/journal.pone.0080662

**Published:** 2013-11-05

**Authors:** Yesenia Vega, Isabel Marques, Sílvia Castro, João Loureiro

**Affiliations:** 1 Departamento de Ciencias Naturales, Universidad Técnica Particular de Loja, Loja, Ecuador; 2 Department of Agriculture (Botany), High Polytechnic School of Huesca, University of Zaragoza, Huesca, Spain; 3 CFE – Centre for Functional Ecology, Department of Life Sciences, University of Coimbra, Coimbra, Portugal; University of Kent, United Kingdom

## Abstract

Hybridization has the potential to contribute to phenotypic and genetic variation and can be a major evolutionary mechanism. However, when hybridization is extensive it can also lead to the blurring of species boundaries and the emergence of cryptic species (i.e., two or more species not distinguishable morphologically). In this study, we address this hypothesis in *Epidendrum*, the largest Neotropical genus of orchids where hybridization is apparently so common that it may explain the high levels of morphological diversity found. Nonetheless, this hypothesis is mostly based on the intermediacy of morphological characters and intermediacy by itself is not a proof of hybridization. Therefore, in this study, we first assessed the existence of hybrids using cpDNA and AFLP data gathered from a large-scale sampling comprising 1038 plants of three species of *Epidendrum* (*E. calanthum*, *E. cochlidium* and *E. schistochilum*). Subsequently, a Bayesian assignment of individuals into different genetic classes (pure species, F1, F2 or backcross generations) revealed that hybrid genotypes were prevalent in all sympatric populations. In most cases, parental species were not assigned as pure individuals, rather consisting in backcrossed genotypes or F_1_ hybrids. We also found that reproductive barriers are apparently very weak in *Epidendrum* because the three species largely overlapped in their flowering periods and interspecific crosses always produced viable seeds. Further, hybridization contributed to enhance floral variability, genome size and reproductive success since we found that these traits were always higher in hybrid classes (F1, F2 and backcrosses) than in pure parental species, and offer an explanation for the blurring of species boundaries in this genus of orchids. We hypothesize that these natural hybrids possess an evolutionary advantage, which may explain the high rates of cryptic species observed in this genus.

## Introduction

The role of hybridization in plant speciation is still one of the most exciting issues in evolutionary biology [1]. Speciation in plants via hybridization is apparently more common than previously thought with several studies suggesting that at least 40% of plant species may have arisen from this process [2-7]. In addition to this constructive role, where hybridization may give rise to new lineages, introgression of genes may also increase genetic variation [8] and genome size [9], but may also lead to the genetic assimilation of their congeners [10].

One major plant group in which hybridization seems to play an important evolutionary role is the genus *Epidendrum* L. (Orchidaceae), although molecular studies actually confirming this hypothesis are scarce [11]. It is the largest neotropical genus of Orchidaceae with almost 1500 species described [12] and hybridization has been suggested to explain the high levels of morphological diversity observed and the existence of cryptic species (i.e., two or more species not distinguishable morphologically). Identifying cryptic species has challenged biologists for a long time, namely because the widely used concept of species boundaries is related with what can be measured morphologically (e.g, Mayr species concept; [13]), and speciation not always produce morphological changes [14]. Hybridization can contribute to the formation of cryptic species because genetically distinct hybrid lineages can look morphologically similar to their parents. However, the rise of relatively fast DNA sequencing techniques has given biologists the power to differentiate among morphologically similar entities [15-17]. 

To evaluate the outcomes of hybridization and whether it is indeed promoting the origin of cryptic species of *Epidendrum*, we selected three species that frequently co-occur, and hypothetically hybridize, in the south of Ecuador: *Epidendrum calanthum* Rchb.f. & Warsz., *E. cochlidium* Lindl. and *E. schistochilum* Schltr. [18]. Hybridization between these species has been hypothesized considering the high floral variability reported and because these species are sometimes hard to recognize in sympatric populations [12]. Using a large-scale sampling including 25 allopatric and 25 sympatric populations, we first ascertained the presence of hybrid plants using data from chloroplast DNA (cpDNA) and amplified fragment length polymorphism (AFLP). Then, we tested if hybridization was promoting variability by comparing the morphology and genome size of different hybrid classes and pure parental species based on the genetic Bayesian assignment of individuals. Finally, flowering asynchrony and the degree of interspecific compatibility were studied to assess their role in the direction of gene flow between *E. calanthum*, *E. cochlidium* and *E. schistochilum*. 

## Methods

### Study system

*Epidendrum calanthum*, *E. cochlidium* and *E. schistochilum* are three terrestrial species of orchids that inhabit open patches and edges of the tropical Ecuadorian forests where there is a moderate to high human impact. Flowers are pink in *E. calanthum*, orange to red in *E. cochiclidum* and yellow to white in *E. schistochilum* [12]. These species are visited by generalist pollinators like diurnal butterflies and syrphid flies and no nectar is produced [19]. Pollination occurs by deception of naïve pollinators, which is quite common in orchids (e.g., [20]). The three species belong to the subgenus *Amphiglottium* and are not closely related since *E. calanthum* and *E. cochlidium* belong to two different phylogenetic groups [21]. The studied species are diploid with 2*n*=28 chromosomes [19]. 

### Plant sampling

A total of 1038 plants from 50 populations (25 sympatric and 25 allopatric populations) were collected. Details of sampling sites are provided in Table S1. The collection of plants was mainly focused in the lower part of Ecuador (province of Loja), where a high variability in floral traits was detected. In each population, individuals were selected with a minimum distance of 10 m along a linear transect covering the length of the population. Each individual was tagged with permanent labels. Flower and leaf tissues from all the individuals were brought back to the laboratory for morphological, genetic and genome size analyzes. Flower and leaf traits were measured in the laboratory in the same day of collection, while the remaining traits were measured in the field. Voucher specimens were deposited at the herbarium of the Universidad Técnica Particular de Loja. For genetic analyses, fresh leaves were stored in silica gel until DNA extraction, whereas for flow cytomety analyses, leaves were stored at 4 °C until analyses (usually within 1-3 days after collection). 

### Admixture analysis and genetic composition of hybrids

Total genomic DNA was extracted using the DNeasyTMPlant Minikit (Qiagen, Hilden, Germany), following the manufacturer’s instructions, and stored at -20 °C. To estimate nuclear admixture proportions and the type of hybrid genotypes (e.g., F_1_, F_2_, backcrosses), 1038 individuals (6-20 per taxon and population) were analyzed using AFLPs (Table S1). The AFLP procedure followed the protocol established by [22]. An initial trial using 16 combinations of primers was conducted on four individuals of each parental species to identify those primers that yielded the highest number of polymorphic peaks among species. The three selected primer combinations were: EcoRI-ACC (FAM)⁄MseI-CAA; EcoRI-AC (FAM)/MseI-CTA and EcoRI-AGG (VIC)⁄MseI-CTC. A reproducibility test was performed by re-extracting DNA from 10% of the samples and repeating the whole AFLP procedure following [23]. Non-reproducible fragments were excluded from analyses. Amplified bands were aligned with the internal size standard using the ABI PRISM Genescan Analysis Software version 3.1 (Applied Biosystems), and the GeneMapper software application (version 3.4; Applied Biosystems) was used to score amplified fragments 100-500 bp in length. Fragments of each primer combination were scored as present (1) or absent (0) and manually corrected.

The genetic composition of hybrids was inferred using the Bayesian clustering method implemented in NEWHYBRIDS version 1.1 beta, which assigns individuals to 6 different classes: 2 pure parental species, F_1_, and F_2_ hybrids and 2 backcrosses with each parental species [24]. Each pair of hybridizing species (e.g., *E. calanthum* x *E. cochlidium*, *E. cochlidium* x *E. schistochilum* and *E. calanthum* x *E. schistochilum*) was analyzed separately using several allopatric populations as reference samples of pure individuals (Table S1). These allopatric populations are far away from the sympatric ones and are composed by only one species of *Epidendrum*. A burnin of 50 000 steps followed by run lengths of 300 000 were used and individuals were classified under a threshold of 0.75.

### DNA extraction and sequencing

A pilot study was performed to find the most variable DNA sequences among eight different chloroplast DNA markers. From those, the following chloroplast DNA regions were identified as the most suitable: *trn*L-*trn*F (c and f [25]:), rps16 (rps16F and R [26]:), *psb*A- *trn*H ([27])and rpl16 (F71 and R1516 [28]:). These regions were sequenced for a representative group of 304 plants (2-5 individuals per population; Table S1). Primer sequences and PCR conditions were obtained from the literature (see references above). All PCR products were purified using UltraClean™PCR Clean-up™Kit (MoBio Laboratories, Inc., Carlsbad, CA, USA) according to the manufacturer’s protocol. Purified PCR products were sequenced in both directions on a 3730 DNA ANALYZER (Applied Biosystems, Foster City, CA, USA). Sequence alignment was performed manually using BioEdit 7.0.0. DnaSP version 3 [29]) was used to characterize DNA polymorphism. The four chloroplast matrices were concatenated and a parsimony network was constructed using TCS version 1.21 [30], with gaps treated as missing data and with a 95% connection limit.

### Floral morphologic variability

To evaluate if hybridization indeed promotes floral variability, the individuals taken for the genetic study and therefore previously assigned as pure species or hybrids (F_1_, F_2_ or backcrosses) were also characterized morphologically (*N* = 1038 plants; Table S1). Measurements were made with a digital caliper accurate to the nearest 0.01 mm. Twenty nine morphological characters considered important in the identification of *Epidendrum* species [12]were measured, including six vegetative and 23 floral characters (Table S2). At the end, 13 characters (seven floral characters plus all the vegetative ones; Table S2) were eliminated from the analyses since they showed no significant differences between species (*P* > 0.05). In order to avoid redundancy in the data set, four characters showing high correlation coefficients with the length of the petal (R > 0.98, *P* < 0.05) were removed from the analyses, resulting in a total final matrix of 12 characters (Table S2). This matrix is available upon request. A principal component (PC) analysis of the log-transformed variables was performed to evaluate morphological variation among species. Normality was previously tested with the Kolmogorov-Smirnov test. To facilitate the interpretation of the multivariate pattern described by the PC analysis and maintain at the same time the orthogonality in the data set, the varimax rotation was used [31]. The morphological components of the PC analysis (i.e., combinations of morphological variables) that presented eigenvalue variances greater than one were then identified and used to explore the relationship between morphology and hybridization through a multivariate analysis of variance (MANOVA). All statistical analyses of this and of the following experiments were performed with R 2.11.0 [32].

### Genome size variation

A total of 158 individuals from 30 populations were used, representing 2-6 individuals per population and comprising 72 hybrids and 86 pure parental individuals (according with the hybrid assignment using NEWHYBRIDS; Table S1). Nuclei were released after co-chopping 5 cm^2^ of fresh leaf tissue of *Epidendrum* sp. together with 0.5 cm^2^ of fresh leaf tissue of *Pisum sativum* (internal reference standard with 2C = 8.76 pg; [33]) with a sharp razor blade in a Petri dish containing 1 ml of WPB buffer [34]. The nuclear suspension was recovered and filtered through a 50-µm nylon filter to remove cell fragments and large debris. Nuclei were stained with 50 mg.ml^-1^ propidium iodide (Fluka, Buchs, Switzerland), and 50 mg.ml^-1^ RNase (Sigma, St Louis, MO, USA) was added to the nuclear suspension to prevent staining of double-stranded RNA. Five minutes after staining, the relative fluorescence intensity of at least 3000 nuclei was analyzed in a Partec CyFlow Space flow cytometer (Partec GmbH., Münster, Germany), equipped with a green solid state laser for PI excitation, using the FloMax software (Partec GmbH). The G_0_/G_1_ peak of the standard was set to channel 100, and then the amplification system was set to a constant voltage and gain throughout the experiment. The resulting histograms were evaluated and the genome size of each sample was determined by multiplying the sample/standard ratio with the genome size of the standard. As a quality control, only when CV values of G_0_/G_1_ peaks were below 5% the analyses were saved; otherwise sample preparation was repeated. The mean and standard deviation of the mean (SD) of each sample were calculated. The normality of the distribution of genome size of all samples was assessed using the Kolmogorov-Smirnov test. Differences of genome size between hybrid classes were evaluated using analysis of variance (one-way ANOVA). In those cases in which ANOVA revealed significant differences, the Tukey HSD post-hoc test was performed. 

### Flowering phenology

In order to determine the degree of flowering overlap between species, three plots of 40 m^2^ were randomly established in six sympatric populations: two of *E. calanthum* and *E. cochlidium* (POP29 and POP34), two of *E. cochlidium* and *E. schistochilum* (POP42 and POP45), and two of *E. calanthum* and *E. schistochilum* (POP38 and POP39). Plots were placed evenly spaced along each population covering its length, but only considering pure-individuals (based on the results showed here and on individuals examined genetically during previous studies; [19]). Phenology was assessed throughout the flowering period of the three species, from the 13^th^ October 2011 to 1^st^ of March 2012. Within each plot, all flowers were censused each two days in a total of 505 individuals of *E. calanthum*, 1116 of *E. cochlidium* and 406 of *E. schistochilum*. Since preliminary analyses showed no significant differences between plots (*P* > 0.05), phenological data from the three plots were pooled within each population. The following flowering variables were calculated, in Julian dates, for each population: (1) onset, the date of the first flower opening; (2) termination, the date of senescence of the last flower; (3) peak, the date when the maximum number of open flowers was registered; (4) duration, the number of days the population remained in bloom; and (5) overlap between species, the percentage of days that two species flower simultaneously. To meet the assumptions of normality, variables were square-root transformed before analysis. To determine the effect of species, population and their interaction on the flowering parameters a univariate General Linear Model (GLM) was used. 

### Interspecific crossability

To determine the level of crossability between species, experimental pollinations were carried out in nine populations, the six sympatric populations used to assess flowering phenology plus three allopatric ones (POP1, POP12 and POP21; Table S1). As described above, only pure individuals that were assigned as such in this or in previous studies were used in this experiment. Plants were bagged with a 1-mm nylon mesh prior to flowering to exclude pollinators, and the following treatments were performed: (1) intraspecific cross-pollination, i.e., crosses between different individuals of the same species from the same population, and (2) interspecific cross-pollination, i.e., crosses between individuals from different species from the same population. Pollinations were performed in both directions by removing pollinia with a plastic toothpick and placing them on the stigmas of other individuals. Reproductive success of open flowers was also followed in these populations. For that, a total of 50 randomly selected flowers (1 flower per individual) were used per population and treatment. Flowers were then monitored for fruit set after anthesis. All mature fruits were collected and seeds were subsequently placed in a 1% solution of triphenyl tetrazolium chloride and stored for 24h at 30 °C to evaluate seed viability. Per fruit, 250 seeds were observed under an optical microscope (100x magnification) and the percentage of viable seeds was calculated. Fruit set and seed viability were log- and square-root transformed, respectively. The effects of treatments on fruit set and seed viability were tested with a GLM, with pollination treatment and populations as fixed factors, and individuals as a random-effect factor. In addition, reproductive success (fruit set and seed viability) was compared between sympatric and allopatric populations using a one-way ANOVA. 

## Results

### Genetic composition of the hybrids

**Figure 1:**
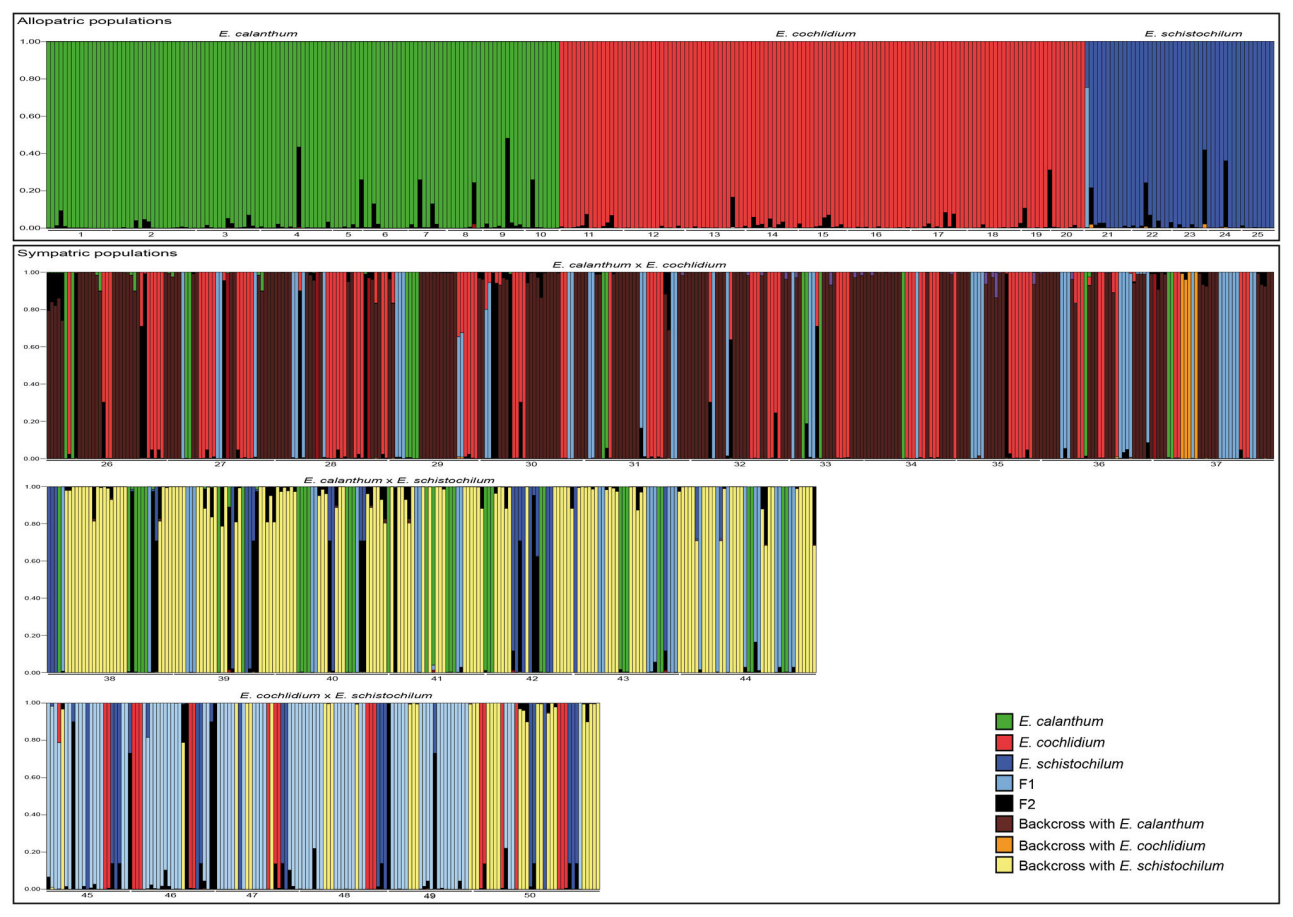
Posterior probabilities (*q*) for all analyzed plants by NEWHYBRIDS (*N*= 1038 plants). Each vertical bar represents an individual plant. The proportion of colour in each bar indicates the assignment probability of the individual according with the different genetic classes. See Figure 1 for details of geographical position of populations.

Based on AFLP data, Bayesian assignment of individuals indicated that the allopatric populations of *E. calanthum*, *E. cochlidium* and *E. schistochilum* were generally composed of purebreds since only one individual (*E. schistochilum*) was assigned as F1 genotype (Figure 1). However, hybrid genotypes were predominant in all sympatric populations since an hybrid status was assigned to 74.9% of the individuals in these populations (550 out of 734; Figure 1). In detail, in the populations of *E. calanthum* and *E. cochlidium*, 72.8% of the parental species sampled were identified as hybrids, whereas, 77.9%, and 75.6% of hybrids were detected, respectively.

Nevertheless, a wide variation was detected in the genetic composition of populations (Figure 1). Backcrosses with *E. calanthum* were predominant in the sympatric populations of *E. calanthum* and *E. cochlidium* (54.7% of individuals), while backcrosses with *E. schistochilum* were abundant when it occurred in sympatry with *E. calanthum* (58.1% of individuals; Figure 1). F_1_ genotypes prevailed in the sympatric populations of *E. cochlidium* and *E. schistochilum* populations (48.1% of individuals). Despite being present in all populations, F_2_ hybrids occurred in low proportions (<5.9% of individuals).

### Chloroplast diversity of parental species and hybrids

The aligned matrix of the four chloroplast regions (*trn*L-*trn*F, rps16, *psb*A- *trn*H and rpl16) had 3616 bp, and all the 247 variable sites (7%) were parsimony-informative. Sequences statistics and GenBank accession numbers are given in Tables S3 and S4, respectively. The hybrids showed the highest levels of nucleotide variability, while the lowest levels were recorded in *E. schistochilum* (Table S3). 

**Figure 2:**
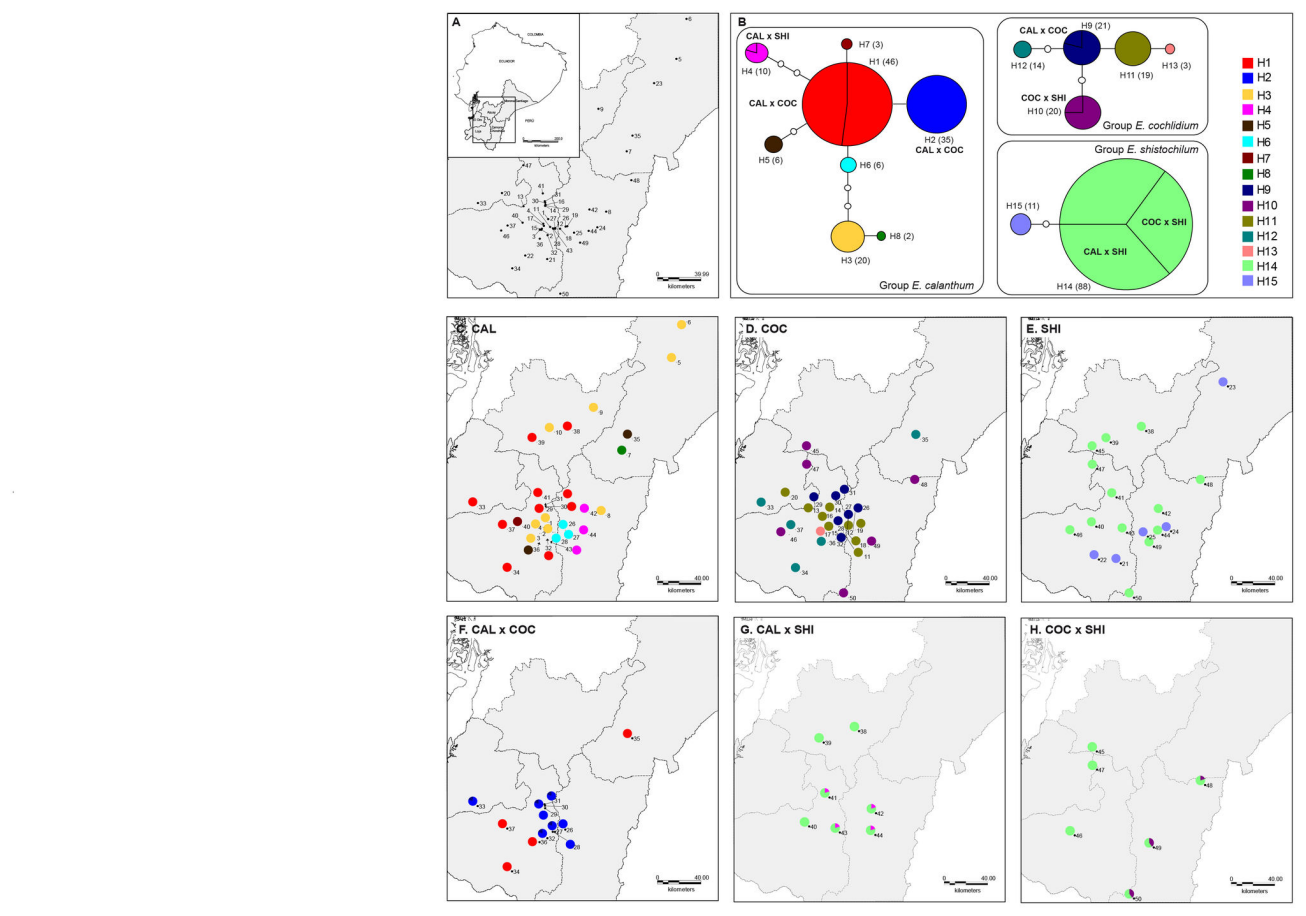
Patterns of haplotype variation in three hybridizing species of *Epidendrum* (*N* = 304 plants). A) Statistical parsimony network of plastid haplotypes based on sequences from four chloroplast regions (*trn*L-*trn*F, rps16, *psb*A- *trn*H and rpl16), a circle’s size being proportional to the haplotype frequency. Small empty circles represent single mutational steps. B) Geographic distribution of plastid haplotypes.

The TCS analysis revealed 15 haplotypes grouped in three unconnected networks (Figure 2b). One network grouped all individuals of *E. calanthum* in seven haplotypes plus an exclusive one for some of the *E. calanthum* x *E. cochlidium* hybrids (H2). The second network grouped all individuals of *E. cochlidium* in 5 haplotypes and the third network grouped all individuals of *E. schistochilum* in 2 haplotypes. H1 and H14 were predominant in the case of *E. calanthum* and *E. schistochilum*, respectively.

Among hybrid plants, 83.3% of *E. schistochilum* x *E. cochlidium* hybrids and 93.9% of *E. schistochilum* x *E. calanthum* shared the haplotype H14 with *E. schistochilum*. The remaining hybrid plants had the same haplotype as *E. cochlidium* (H10) and *E. calanthum* (H4), respectively. In the case of *E. calanthum* x *E. cochlidium* hybrids, most of them had the exclusive haplotype H2 (56%) while the remaining individuals were grouped with *E. calanthum* (36%; H1) or *E. cochlidium* (8%; H9 Figure 1). The geographic distribution of haplotypes is presented in Figure 2(c-h). Although the distribution of haplotypes does not show a geographic pattern, most allopatric populations have only one haplotype (H14 for *E. schistochilum*, H3 for most *E. calanthum* and H11 for most *E. cochlidium* populations), while sympatric populations have a wider diversity of haplotypes. 

### Floral morphologic variability

Populations were usually characterized by a high intraspecific floral variability (Figure S1). The PC analyses identified two axes with eigenvalues > 1 for *E. calanthum*, *E. cochlidium* and *E. schistochilum* (Table S5) accounting for 75.3%, 68.2% and 77.2% of the observed morphological variation, respectively. In the three species, the main component (PC1, accounting for 34.2% of variance in *E. calanthum*, 30.1% in *E. cochilidium* and 38.9% in *E. schistochilum*) summarized floral traits such as the column, lateral and central lobe of the lip and the size of the callus, whereas component PC2 reflected morphological traits of the dorsal sepal and petal (Table S5). 

**Figure 3:**
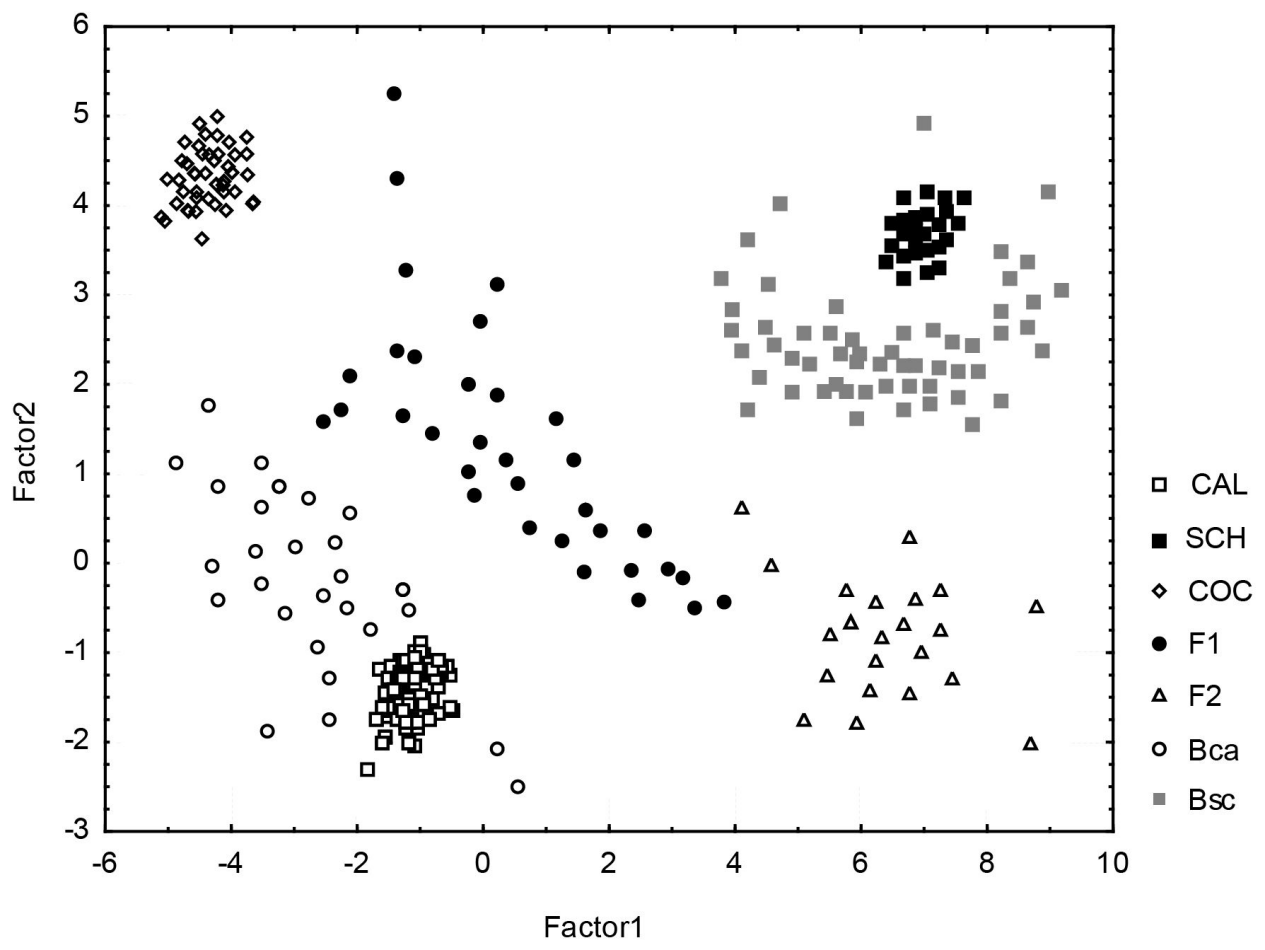
Scatter plot of the principal component analysis (PCA) of morphological variations in the pure parental species, *Epidendrum* *calanthum* (CAL), *E*. *schistochilum* (SCH), and *E*. *cochlidium* (COC) and the hybrid generations (F_1_, F_2_ and the two backcrossed hybrids, Bca and Bsc) grown in their natural habitats. Genetic groups were gathered using NEWHYBRIDS.

When species were analyzed together considering the genetic groups gathered in NEWHYBRIDS, all classes were separated, although the distribution of hybrid groups was always wider than that of pure parental species (Figure 3). Hybrid classes had significant effects on the main morphological score, PC1 (MANOVA: *E. calanthum*: *F*_*4,137*_ = 0.310, *P* < 0.001; *E. cochilidium*: *F*_*4,145*_ = 0.452, *P* < 0.001; *E. schistochilum*: *F*_*4,128*_ = 1.241, *P* < 0.001), i.e., this morphological score increased in all hybrid classes in comparison to pure parental species (Figure S2).

### Genome size variation

Mean genome size estimated in pure parental individuals was 3.72 ± 0.08 pg/2C in *E. calanthum*, 3.98 ± 0.10 pg/2C in *E. cochlidium* and 3.96 ± 0.04 pg/2C in *E. schistochilum*. Genome size showed a significant interspecific variation since values were lower in *E. calanthum* than in the remaining two species (ANOVA test, *F*_2,77_ = 80.860, *P* < 0.001 followed by a Tukey test *P* < 0.001). 

**Figure 4:**
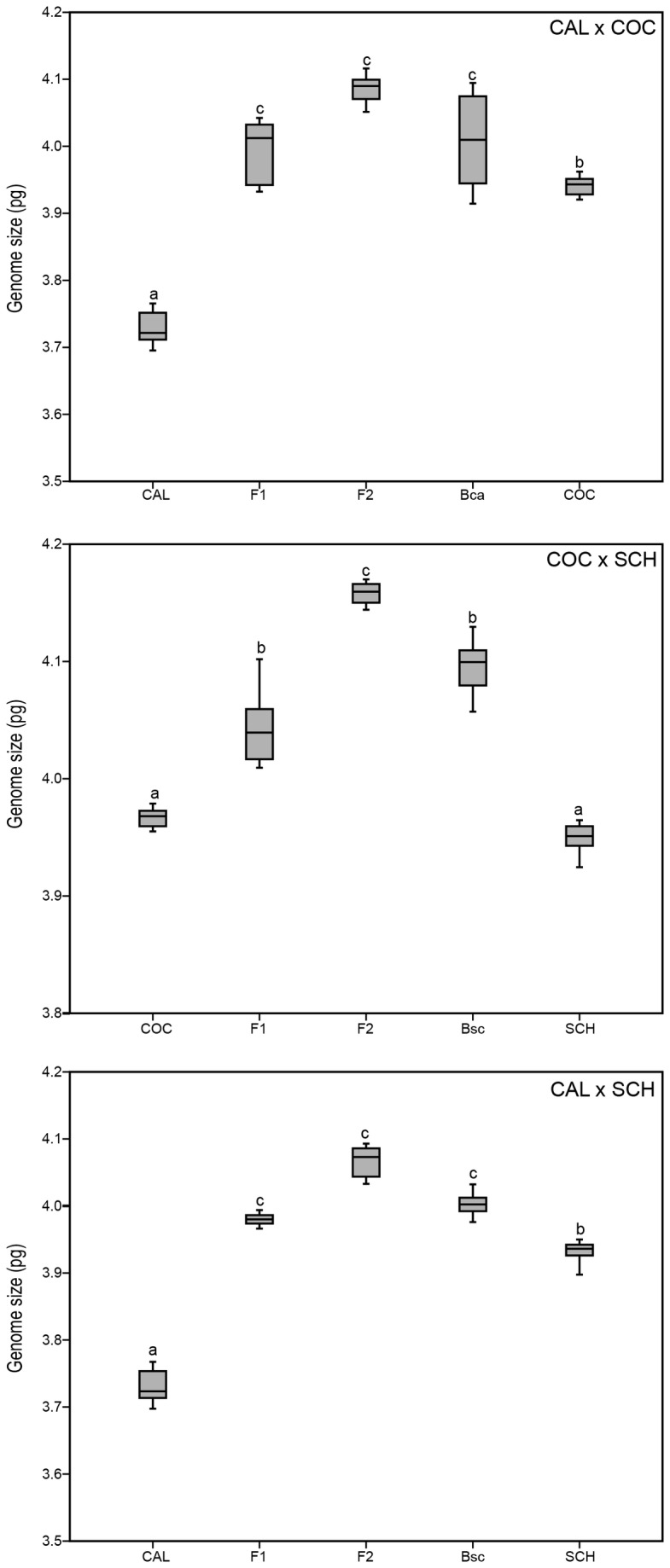
Genome size obtained of the three pairs of hybridizing species of *Epidendrum*: *E*. *calanthum* x *E*. *cochlidium*; (A); *E*. *cochlidium* x *E*. *schistochilum* (B); *E*. *calanthum* x *E*. *schistochilum* (C). CAL = *E*. *calanthum*; COC = *E*. *cochlidium*; SCH = E. schistochilum according with genetic groups detected previously by NEWHYBRIDS. Values are expressed in pg (*N* = 158 plants).

However, genome size of all hybrid classes were always higher than those of the parental species (Figure 4): hybrids vs. *E. calanthum* and *E. cochlidium* (*F*_2,47_ = 283.319, *P* < 0.001), hybrids vs. *E. schistochilum* and *E. cochlidium* (*F*_2,37_ = 55.546, *P* < 0.001) and hybrids vs. *E. calanthum* and *E. schistochilum* (*F*_2,37_ = 94.618, *P* < 0.001). The maximum increase in genome size was always recorded in F_2_ hybrids (Figure 4).

### Flowering phenology

*Epidendrum cochlidium* always started the flowering period earlier than *E. calanthum* (~21 days earlier) and *E. schistochilum* (~5 days earlier), although the flowering peak matched in the three species (Table 1). The flowering period of *E. calanthum* and *E. schistochilum* completely overlapped with that of *E. cochlidium*, which had a wider phenological period (Table 1). 

**Table 1:** Phenological parameters (mean ± SD) of *Epidendrum* *calanthum* (CAL), *E. cochlidium* (COC) and *E. schistochilum* (SCH) measured in six sympatric populations: two with *E. calanthum* and *E. cochlidium* (POP29 and POP34), two with *E. calanthum* and *E. schistochilum* (POP38 and POP39) and two with *E. cochlidium* and *E. schistochilum* (POP42 and POP45).

**Phenological parameters**	**POP29**		**POP34**		**POP38**		**POP39**		**POP42**		**POP45**		
	**CAL**	**COC**	**CAL**	**COC**	**CAL**	**SCH**	**CAL**	**SCH**	**COC**	**SCH**	**COC**	**SCH**
	*N* = 112	*N* = 221	*N* = 99	*N* = 201	*N* = 101	*N* = 82	*N* = 95	*N* = 84	*N* = 237	*N* = 81	*N* = 215	*N* = 80
**Onset**	38.2 ± 5.4	17.1 ± 1.3	39.1 ± 4.3	18.5 ± 1.8	31.7 ± 2.5	26.4 ± 2.8	33.5 ± 1.9	24.7 ± 2.1	21.4 ± 1.3	26.3 ± 1.5	22.8 ± 1.1	28.4 ± 2.2
**Peak**	61.3 ± 4.2	59.4 ± 3.8	63.4 ± 3.7	61.5 ± 2.9	52.7 ± 1.8	45.7 ± 2.1	55.3 ± 1.2	48.2 ± 1.8	52.7 ± 2.1	54.1 ± 2.4	49.3 ± 2.3	51.7 ± 1.9
**Termination**	110.3 ± 3.2	145.2 ± 3.8	108.7 ± 2.1	153.2 ± 3.1	103.2 ± 4.8	80.3 ± 3.5	105.6 ± 5.7	82.4 ± 2.6	131.5 ± 4.7	75.6 ± 3.8	134.7 ± 5.8	72.1 ± 2.9
**Duration**	72.4 ± 2.5	128.9 ± 4.9	69.6 ± 1.8	134.7 ± 3.4	72.6 ± 3.1	54.3 ± 1.8	72.1 ± 4.8	57.7 ± 4.6	110.3 ± 2.8	49.8 ± 3.1	111.9 ± 3.2	43.7 ± 2.9
**Overlap**	100%	56.30%	100%	55.21%	68.06%	90.74%	69.94%	94.88%	44.50%	100%	42.28%	100%

Onset, peak, termination and duration of the flowering period are given in Julian date format (day 1 = October 2011). *N* = number of flowers recorded.

In the case of sympatric populations of *E. calanthum* and *E. schistochilum*, the latter species always bloomed first and had a shorter flowering period than that of *E. calanthum*, overlapping in 90.7%-94.9% of the days (Table 1). The flowering peak usually occurred one week earlier in *E. schistochilum* and flowering finished approximately 21 days earlier than in *E. calanthum* (Table 1). Statistically significant differences among species and populations were detected for all phenological parameters (Table S6).

### Interspecific crossability

**Figure 5:**
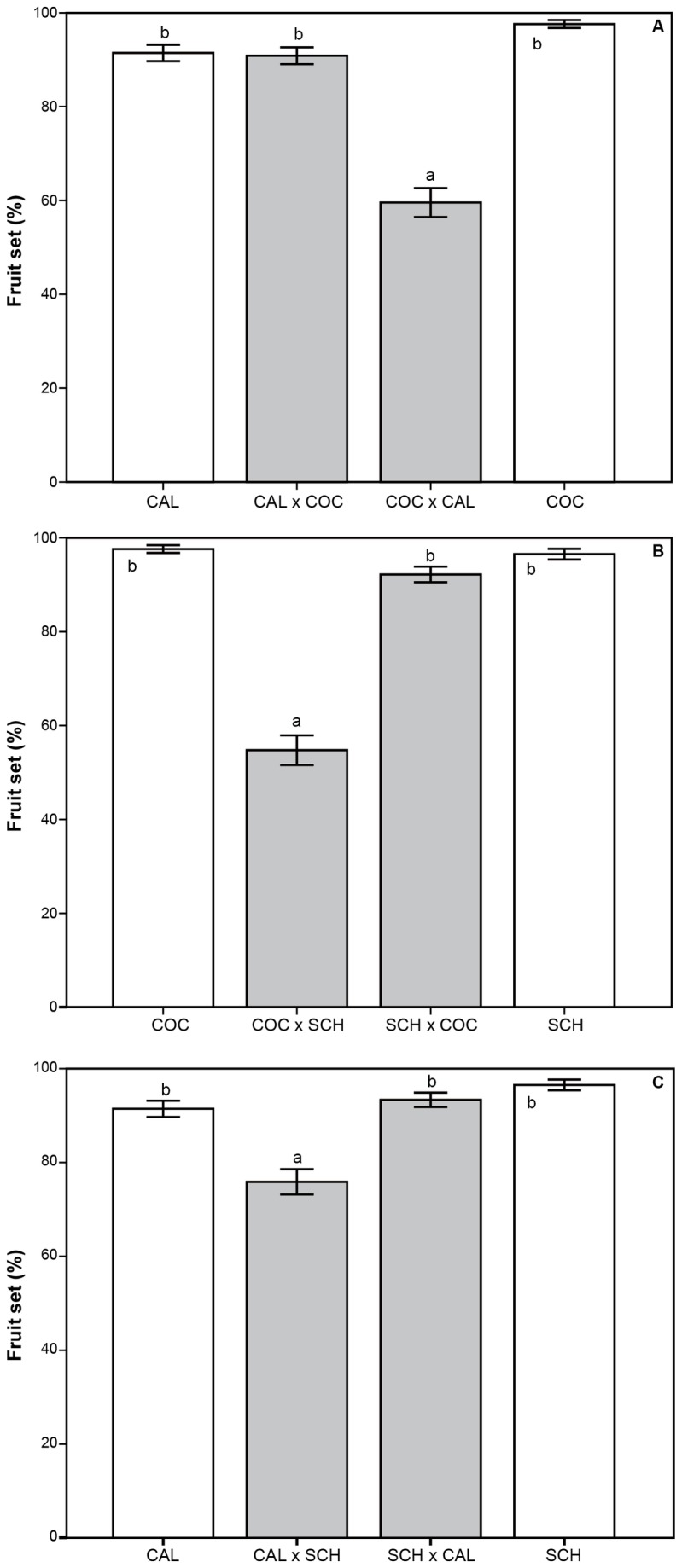
Mean fruit set after experimental crosses within (white bars) and between (grey bars) the studied species considering three pairs of hybridizing species of *Epidendrum*: *E*. *calanthum* x *E*. *cochlidium*; (A); *E*. *cochlidium* x *E*. *schistochilum* (B); *E*. *calanthum* x *E*. *schistochilum* (C). Values indicate means ± SD (*N* = 100 plants/cross). The first letters indicate the identity of the mother species: CAL = *E*. *calanthum*; COC = *E*. *cochlidium*; SCH = *E*. *schistochilum*. Crosses with the same letter did not differ significantly (*P* > 0.05).

Experimental pollinations revealed that the three species can set a high quantity90% to 96% of fruits in intraspecific crosses (Figure 5). Nevertheless, results from interspecific crossability ranged between 56% and 94%, being dependent on the species that acted as pollen recipient (GLM: F_5,1490_ < 0.0001). When *E. calanthum* or *E. schistochilum* received pollen from *E. cochlidium*, no differences were detected between interspecific and intraspecific crosses although fruit set was lower in the opposite crosses (Figure 5a,b). A similar breakdown in the formation of interspecific fruits was found when *E. calanthum* received pollen from *E. schistochilum*, but not in the opposite cross (Figure 5c). No differences were found between populations (GLM: F_4,245_ = 0.085, *P* = 0.987). The number of seeds per capsule was 281 ± 141 (mean ± SD), of which 55.3 ± 15.6% (mean ± SD) were viable seeds. No significant differences in seed viability were found between species (F_4,245_ = 0.131, *P* = 0.971) and populations (F_4,245_ = 0.113, *P* = 0.978). 

### Reproductive success in natural populations

In natural populations, fruit set of open flowers varied between 30% and 52% in *E. calanthum*, between 34% and 56% in *E. cochlidium* and between 22% and 44% in *E. schistochilum*. Results varied significantly between the three species (GLM: F_2,747_ = 3.548, *P* = 0.029; Table 2). In addition, fruit set also varied between populations since it was always higher in sympatric than in allopatric populations (F_1,248_ = 6.110, *P* = 0.014; Table 2). In the case of seed viability, no differences were found between species (53.3 ± 21.1%; F_1,249_ = 6.315, *P* = 0.891) or populations (F_1,249_ = 5.89, *P* = 0.781). 

**Table 2:** Average fruit set in four natural sympatric populations of *Epidendrum calanthum*, *E. cochlidium* and *E. schistochilum* plus three alopatric populations.

**POP**	***E. calanthum***	**POP**	***E. cochlidium***	**POP**	***E. schistochilum***
1	0.30 ± 0.46ª	12	0.34 ± 0.47ª	21	0.22 ± 0.41ª
29	0.51 ± 0.50^c^	29	0.55 ± 0.49^c^	38	0.41 ± 0.49^b^
34	0.52 ± 0.50^c^	34	0.56 ± 0.50^c^	39	0.40 ± 0.50^b^
38	0.38 ± 0.50^b^	42	0.50 ± 0.51^b,c^	42	0.44 ± 0.51^b^
39	0.40 ± 0.49^b^	45	0.52 ± 0.51^b,c^	45	0.42 ± 0.50^b^

Mean ± SD (*N* = 50 plants per species and population). Superscripts indicate comparisons between treatments using Scheffe test. Treatments with the same letter do not differ significantly (P > 0.05).

## Discussion

### Extensive past and recent asymmetric hybridization gives rise to divergent hybrid zones

The results of our study provide evidence that *E. calanthum*, *E. cochlidium* and *E. schistochilum* frequently hybridize at sites where they co-occur. Hybrid genotypes were prevalent in all sympatric populations, where almost 75% of the individuals were assigned an hybrid status. Backcrosses towards *E. calanthum* were predominant when this species co-occurred with *E. cochlidium*, but when it co-occurred with *E. schistochilum*, backcrosses towards the latter species prevailed. F_1_ hybrids were only frequent in the sympatric populations of *E. cochlidium* and *E. schistochilum*. This wide variety of results implies the existence of divergent hybrid zones and challenges the widely view of bimodal (where populations are generally composed by individuals genetically similar to one or the other parental species; [35]) vs. unimodal hybrid zones (where intermediate hybrid genotypes predominate; [36]). As demonstrated here, genotype frequencies may vary considerably between contact zones allowing different situations to occur. 

Furthermore, the presence of recombinant hybrid classes like backcrosses suggest that natural hybridization is not recent. A low frequency of F_1_ individuals is known from other hybrid zones and is usually associated with strong assortative mating [37,38]. For instance, in a hybrid zone between Louisiana *Iris* species, F_1_ hybrids are extremely rare, but backcrosses are relatively abundant as they exhibit a high fitness in different types of habitats [39]. Nonetheless, the existence of an exclusive hybrid haplotype in some of the hybridizing populations of *E. calanthum* × *E. cochlidium*, together with the predominant presence of F_1_ hybrids suggest a different process of hybridization in these populations with the presence of past and current gene flow between these species. 

Although hybridization can occur in both directions as revealed by our crossing experiments, hybrid formation was highly asymmetric. Results based on cpDNA revealed that *E. schistochilum* was the mother species of almost all hybrids when this species occurred in sympatry with *E. calanthum* and *E. cochlidium*, while *E. calanthum* was the predominant mother of the natural hybrids generated with *E. cochlidium*. This asymmetric hybrid formation was also supported by the different siring abilities after controlled interspecific crosses, which followed a pattern similar to the one observed with cpDNA. Highly asymmetric hybrid formation is not unusual in nature [40] and can be explained by asymmetric gene flow. For instance, *E. calanthum* and *E. schistochilum* flowered entirely at the same time than *E. cochlidium*, which means that they can receive interspecific pollen during the entire flowering period. By contrary, *E. cochlidium*, can only receive conspecific pollen in some days of its flowering period. The same applies in the populations of *E. calanthum* and *E. schistochilum*, where the latter species have a shorter flowering period overlapping entirely with that of *E. calanthum*. 

### Reproductive isolation between sympatric *Epidendrum* species

None of the reproductive barriers studied here seems to contribute highly to the isolation of these species, supporting the extensive interspecific gene flow observed between the three studied species. Flowering asynchrony, which is one the strongest pre-zygotic barriers between species [41,42]contributes very little to the isolation between *Epidendrum* species since there was always a high proportion of individuals flowering simultaneously. As there is no apparent mechanism of floral or ethological isolation, as observed in many other co-occurring deceptive pollinator orchids [43,44], naïve generalist pollinators visit these co-occurring species of *Epidendrum* indiscriminately, contributing to the formation of hybrid embryos [19]. The high degree of interspecific compatibility between *E. calanthum*, *E. cochlidium* and *E. schistochilum*, demonstrated by the crossing experiments and by the fact that seeds are fertile also supports a lack of pollen-pistil incompatibility and post-zygotic genomic incompatibility. 

Besides the reproductive barriers studied here, the three species did not display different habitat or ecological preferences since they can be found growing side by side in the same environments. Also, although we did not analyze the effect of scent as a reproductive barrier, the fact that several generalist insects were frequently observed pollinating congeneric species [19], suggests that floral odors do not act as a barrier to promote specific pollinator attraction in *Epidendrum*, as opposed to what has been described in other orchids [43,45]. The fact that a large proportion of backcrosses were detected in the natural populations studied, together with the presence of F_2_ hybrids, suggest that the commonly reported hybrid pollen sterility [46,47] does not apply to *Epidendrum*, at least in the early hybrid generations. Differences in genome size are also not enough to prevent gene flow between *E. calanthum*, *E. cochlidium* and *E. schistochilum*. 

### Phenotypic, reproductive and genome size consequences of hybridization

It is widely assumed that hybridization can induce rapid genome size changes, including the gain or loss of DNA, although comparisons of DNA amounts between hybrids and their parents are actually limited to a few cases in the literature (e.g., [9]). Generally, one would expect that DNA contents of hybrids would be intermediate to those of their parents like demonstrated by [48]using herbarium vouchers. Nevertheless, all hybrid classes studied here exhibit significantly higher nuclear DNA contents than their parental species, with the magnitude of genome size increase being independent of the maternal species. Similar results were also reported in *Helianthus* homoploid hybrids, with the increase in DNA amounts occurring independently and repeatedly, although a maternal effect was detected in this case [9]. Here, we observed an increase in genome size in only two generations, with backcrosses between F_1_ individuals and parental species presenting a higher variability in genome size and intermediate genome sizes between F_1_ and F_2_ hybrids. Previous hybridization studies in *Zea mays* [49], *Narcissus* [50]and in animals (e.g., [51]) have already evidenced that changes in genome size can occur in a single generation through transposon replication and/or gain of chromosome regions, such as tandem repeats. The functional and evolutionary effects of a higher genome size in the hybrids are still unknown, but considering that hybrid genotypes were prevalent in the contact sites and that the fruit set was higher in sympatric populations than in allopatric ones, it seems that hybrids may have significant short-time selective advantages over their parents.

Hybridization can also contribute to an increase in morphological diversity of the populations as a result of segregation and recombination between the parental genomes [52,53]. Previous works have shown that hybrids are usually a complex mosaic of both parental morphological characters rather than just intermediate phenotypes, and a large proportion of first and later generation hybrids exhibit extreme or novel characters [54]. High morphological variability in *Epidendrum* hybrids was also observed in our study (Figure 3) and it might be responsible for the attraction of additional species of pollinators contributing to a higher reproductive success in these populations (Vega, *unpublished data*). 

### Mechanisms of speciation in *Epidendrum* and the origin of cryptic species

One of the most interesting evolutionary aspects of hybridization is the fate of hybrids once they are formed. In the absence of reproductive barriers, successful hybridization and introgression may threaten the existence of their parents through genetic assimilation or demographic displacement [10]. Our results show that the absence of reproductive barriers enables hybridization between sympatric species of *Epidendrum*, and hybrids become dominant in the contact sites. Although fitness of natural hybrids of *Epidendrum* was not quantified, the prevalence of hybrid genotypes and higher reproductive success recorded in sympatric populations suggest that hybrids are fertile and may even outperform parental species in the contact sites, as observed in other plant groups [55,56]. 

Without the existence of strong reproductive barriers, gene flow between species may easily usurp ovules that would be used in conspecific pollinations. Vegetative propagation observed in these species [19] may also enable their persistence in sympatric populations. Hybrids can also serve as bridges for gene flow with parental species, producing backcrossed lineages that enhance genetic and floral diversity and overall fitness [39,57]. This hypothesis can also be considered in *Epidendrum* since backcrosses are predominant in most populations (results herein and in [11]). Since none of the reproductive barriers contributes effectively to isolation, hybrid genotypes are frequent and prevalent whenever more than one species of *Epidendrum* occurs together, which offers an explanation for the high levels of cryptic species found in this genus of orchids. Nonetheless, an important question to bear in mind is whether there are still different species in these sympatric populations. As interspecific gene flow is frequent and the new lineages were able to backcross, species cohesion is difficult to accept in orchids. Wherever lays the definition of species boundaries, it is no doubt questionable in orchids making it difficult to establish natural entities.

The importance of hybridization as a source of genetic novelties in orchids is far from being resolved due to the huge amount of orchids described. No generalizations can be performed even in *Epidendrum* since more than 1500 species have been recognized [12], and molecular studies are unavailable for almost all hybridizing populations. Our results support a lack of strong pre- and post-zygotic barriers, since viable hybrids were easily produced and therefore, hybridization was a general outcome in all sympatric populations. However, in a previous published study in *Epidendrum*, some reproductive barriers have been found since F_1_ hybrids and backcross individuals did not produce fruits when they acted as pollen donors [11]. Also, in contrast to our results, the presence of hybrids and backcrossed individuals was smaller, and in some cases, parental species were still found to be genetically-pure [11]. Clearly, more studies are needed to understand the importance of hybridization in the diversification of this genus. For instance, the general role of reproductive barriers in these orchids, the influence of habitat types, the fitness of different hybrid classes or even the relationships between parental/hybrids and the mycorrhizal community is far from being resolved and needs further attention in future studies.

## Supporting Information

Figure S1 **Morphological variation of flowers in individuals of *Epidendrum calanthum* in POP 27 (Loja, El Tiro).** Flowers are labeled according with the genetic groups detected by NEWHYBRIDS.
(TIF)

Figure S2 **Box-plot representation of morphological variation in PCA1 according to the genetic groups detected previously by NEWHYBRIDS in three types of sympatric populations of *Epidendrum*: CAL x COC, COC x SCH and CAL x SCH.** CAL = *E. calanthum*; COC = *E. cochlidium*; SCH = *E. schistochilum*. Horizontal lines represent the median, and boxes and whiskers represent the interquartile range and the nonoutlier ranges, respectively.
(PDF)

Table S1 **Populations studied, geographic coordinates and sampling number for morphological, genetic, and flow cytometric analyses.**
(XLSX)

Table S2 **Caracters included in the morphological study of *Epidendrum calanthum*, *E. cochlidium* and *E. schistochilum* and abbreviations used in the text.** *indicates variables that were eliminated from the analyses since they showed no significant differences between species, or because they showed high correlation coefficients (see text for further details).
(DOCX)

Table S3 **Comparative information for the cpDNA (*trn*L-*trn*F, rps16, *psb*A- *trn*H and rpl16) surveyed.** **H**: **number of haplotypes; N_var_: number of variable sites; N_par_: number of parsimony informative sites; H_d_: Haplotype diversity (for each region); π: nucleotide diversity; GC: GC content**.
(DOCX)

Table S4 **GenBank accessions of DNA sequences analyzed in this study.**
(XLSX)

Table S5 **Principal components (PCs, eigenvalues > 1) of morphological traits of *Epidendrum calanthum* (CAL), *E. cochlidium* (COC) and *E. schistochilum* (SCH) measured in 50 natural populations (*N* = 1038 plants).**
(DOCX)

Table S6 **Analysis of variation under a Univariate General Linear Model of four phenological parameters: onset, peak, termination and duration of flowering.** *, **, *** indicates significant differences at *P* = 0.05, *P* = 0.01, and *P* = 0.001 respectively.
(DOCX)
