# Supplementary figures and images for: Outcomes of Extensive Hybridization and Introgression in Epidendrum (Orchidaceae): Can We Rely on Species Boundaries?
Source: PLoS One. 2013 Nov 5;8(11):e80662. doi: 10.1371/journal.pone.0080662 (PMC3818259; doi:10.1371/journal.pone.0080662)

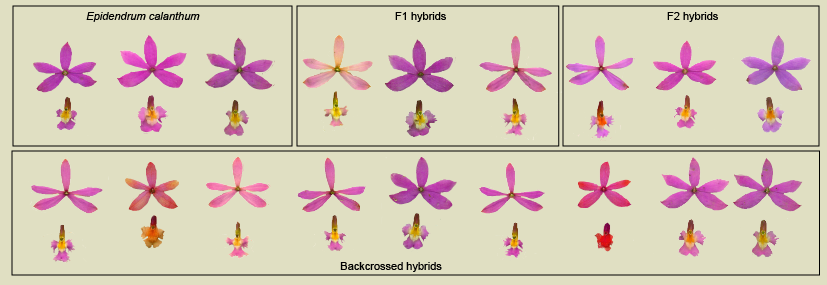

Supplement: Figure S1 — Morphological variation of flowers in individuals of Epidendrum calanthum in POP 27 (Loja, El Tiro). Flowers are labeled according with the genetic groups detected by NEWHYBRIDS. (TIF) [file pone.0080662.s001.tif]

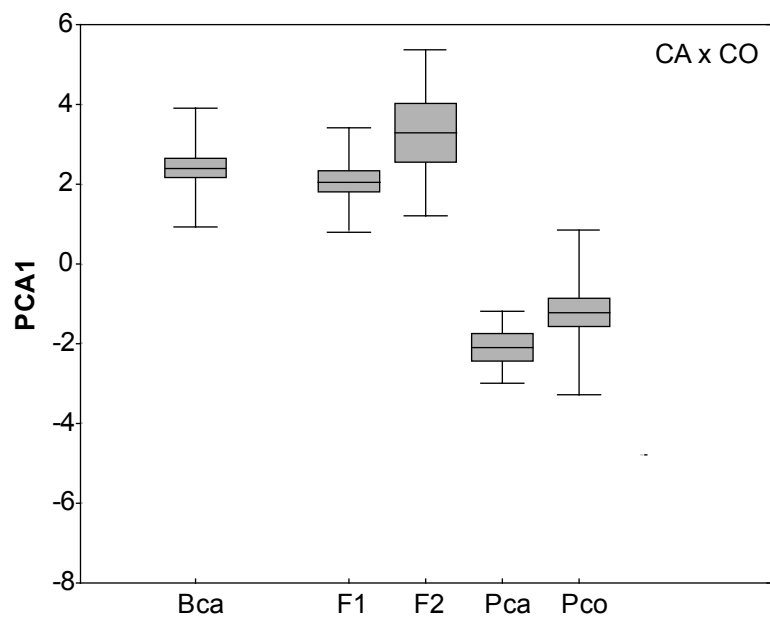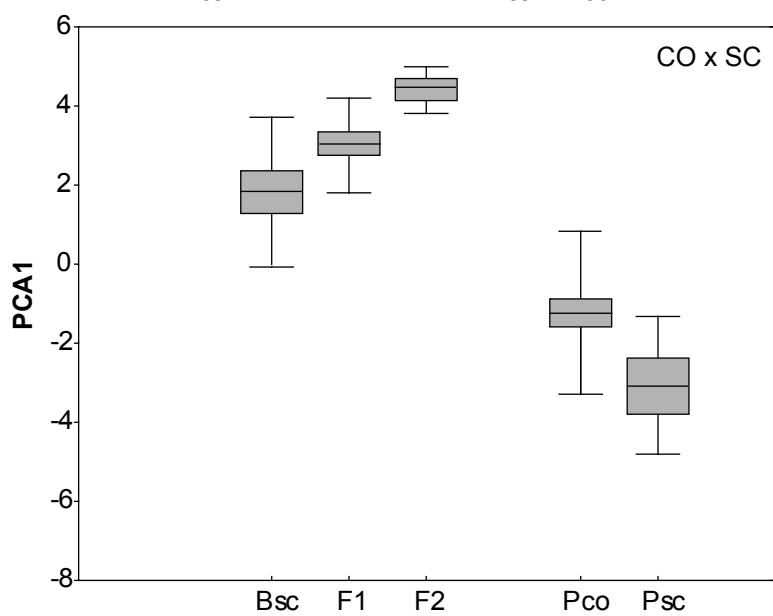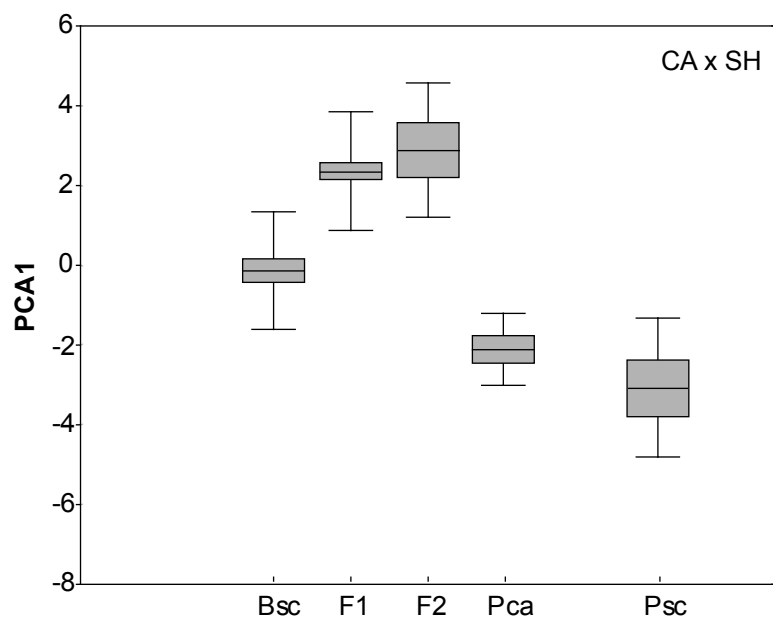

Supplement: Figure S2 — Box-plot representation of morphological variation in PCA1 according to the genetic groups detected previously by NEWHYBRIDS in three types of sympatric populations of Epidendrum: CAL x COC, COC x SCH and CAL x SCH. CAL = E. calanthum; COC = E. cochlidium; SCH = E. schistochilum. Horizontal lines represent the median, and boxes and whiskers represent the interquartile range and the nonoutlier ranges, respectively. (PDF) [file pone.0080662.s002.pdf]
