# Supplementary material for: Outcomes of Extensive Hybridization and Introgression in Epidendrum (Orchidaceae): Can We Rely on Species Boundaries?
Source: PLoS One. 2013 Nov 5;8(11):e80662. doi: 10.1371/journal.pone.0080662 (PMC3818259; doi:10.1371/journal.pone.0080662)
Supplement: Table S2 — Caracters included in the morphological study of Epidendrum calanthum, E. cochlidium and E. schistochilum and abbreviations used in the text. *indicates variables that were eliminated from the analyses since they showed no significant differences between species, or because they showed high correlation coefficients (see text for further details). (DOCX) [file pone.0080662.s004.docx]

**Table S2.** Morphological characters included in the morphological study of *Epidendrum* *calanthum*, *E.* *cochlidium* and *E.* *schistochilum* and abbreviations used in the text. *indicates variables that were eliminated from the analyses since they showed no significant differences between species or variables showing high correlation coefficients (see text for further details).

| **Characters** | **Abbreviation** |
| --- | --- |
| **Floral traits** |  |
| 1. Dorsal sepal length | DS_L |
| 2. Dorsal sepal width | DS_W |
| 3. Lateral sepal length | LS_L* |
| 4. Lateral sepal width | LS_W* |
| 5. Petal length | PT_L |
| 6. Petal width | PT_W |
| 7. Lip length | LA_L* |
| 8. Lip width | LA_W* |
| 9. Column length | CO_L |
| 10. Column width | CO_W |
| 11. Lateral lobe of lip length | LL_L |
| 12. Lateral lobe of lip width | LL_W |
| 13. Central lobe of lip length | CL_L |
| 14. Central lobe of lip width | CL_W |
| 15. Callus of lip length | CA_L |
| 16. Callus of lip width | CA_W |
| 17. Inflorescence length | IN_L* |
| 18. Inflorescence width | IN_W* |
| 19. Inflorescence number | IN_N* |
| 20. Flower number | FL_N* |
| 21. Pedicel length | PE_L* |
| 22. Pedicel width | PE_W* |
| 23. Flower buds number | BD_N* |
| **Vegetative traits** |  |
| 24. Leaf length | LE_L* |
| 25. Leaf width | LE_W* |
| 26. Leaf number | LE_N* |
| 27. Stem length | ST_L* |
| 28. Stem width | ST_W* |
| 29. Stem sheaths number | IN_N* |
