# Supplementary material for: Outcomes of Extensive Hybridization and Introgression in Epidendrum (Orchidaceae): Can We Rely on Species Boundaries?
Source: PLoS One. 2013 Nov 5;8(11):e80662. doi: 10.1371/journal.pone.0080662 (PMC3818259; doi:10.1371/journal.pone.0080662)
Supplement: Table S3 — Comparative information for the cpDNA (trnL-trnF, rps16, psbA- trnH and rpl16) surveyed. H: number of haplotypes; Nvar: number of variable sites; Npar: number of parsimony informative sites; Hd: Haplotype diversity (for each region); π: nucleotide diversity; GC: GC content. (DOCX) [file pone.0080662.s005.docx]

Table S3. Comparative information for cpDNA (*trn*L-*trn*F, rps16, *psb*A- *trn*H and rpl16) surveyed. H: number of haplotypes; N_var_: number of variable sites; N_par_: number of parsimony informative sites; H_d_: Haplotype diversity (for each region); π: nucleotide diversity; GC: GC content.

|  | **H_cp_** | **N_var_** | N_par_ | H_d_ | π | GC |
| --- | --- | --- | --- | --- | --- | --- |
| E. calanthum | 7 | 10 | 10 | 0.775 | 0.00075 | 0.334 |
| *E. cochlidium* | 5 | 6 | 6 | 0.778 | 0.00053 | 0.325 |
| *E. schistochilum* | 2 | 2 | 2 | 0.390 | 0.00022 | 0.320 |
| *E. calanthum x E. cochlidium* | 3 | 140 | 140 | 0.551 | 0.00618 | 0.332 |
| *E. calanthum x E. schistochilum* | 3 | 222 | 222 | 0.215 | 0.01292 | 0.332 |
| E. cochlidium x E. schistochilum | 2 | 119 | 119 | 0.287 | 0.00949 | 0.321 |
