# Supplementary material for: Outcomes of Extensive Hybridization and Introgression in Epidendrum (Orchidaceae): Can We Rely on Species Boundaries?
Source: PLoS One. 2013 Nov 5;8(11):e80662. doi: 10.1371/journal.pone.0080662 (PMC3818259; doi:10.1371/journal.pone.0080662)
Supplement: Table S5 — Principal components (PCs, eigenvalues > 1) of morphological traits of Epidendrum calanthum (CAL), E. cochlidium (COC) and E. schistochilum (SCH) measured in 50 natural populations (N = 1038 plants). (DOCX) [file pone.0080662.s007.docx]

**Table S5.** Principal components (PCs, eigenvalues > 1) of morphological traits of *Epidendrum* *calanthum* (CAL), *E.* *cochlidium* (COC) and *E. schistochilum* (SCH) measured in 50 natural populations (*N* = 1038 plants).

|  | | | | **CAL** | | | |  | **COC** | | | | |  | **SCH** | | |
| --- | --- | --- | --- | --- | --- | --- | --- | --- | --- | --- | --- | --- | --- | --- | --- | --- | --- |
|  | | | | **PCA 1** | | **PCA 2** | **PCA 3** |  | **PCA 1** | | **PCA 2** | | **PCA 3** |  | **PCA 1** | **PCA 2** | **PCA 3** |
| Eigenvalue | | | | 3.08 | | 2.59 | 1.11 |  | 2.71 | | 2.23 | | 1.20 |  | 3.11 | 2.19 | 1.25 |
| % variance explained | | | | 34.18 | | 28.79 | 12.32 |  | 30.1 | | 24.8 | | 13.33 |  | 38.91 | 24.36 | 13.88 |
| Morphological traits | | | |  | |  |  |  |  | |  | |  |  |  |  |  |
| DS_L | | | | 0.33 | | -0.81* | 0.21 |  | 0.11 | | 0.67* | | 0.14 |  | 0.15 | 0.74* | 0.17 |
| DS_W | | | | 0.42 | | -0.74* | 0.12 |  | -0.01 | | 0.71* | | -0.54* |  | -0.02 | 0.79* | 0.51 |
| PT_L | | | | 0.41 | | 0.19* | 0.78* |  | 0.45 | | -0.21* | | -0.62* |  | 0.24 | 0.26* | -0.25 |
| PT_W | | | | 0.44 | | -0.52* | -0.44 |  | -0.14 | | 0.66* | | 0.59* |  | -0.21 | 0.34* | -0.83* |
| CO_L | | | | -0.63* | | -0.56 | 0.39 |  | -0.80* | | 0.26 | | -0.25 |  | -0.89* | 0.15 | -0.05 |
| CO_W | | | | -0.74* | | -0.20 | 0.22 |  | -0.63* | | -0.32 | | -0.21 |  | -0.71* | -0.21 | -0.34 |
| LL_L | | | | -0.05* | | 0.94 | 0.05* |  | 0.35* | | -0.84 | | 0.01 |  | 0.31* | -0.66 | -0.01* |
| LL_W | | | | -0.81* | | 0.51 | -0.28* |  | -0.59* | | -0.59 | | 0.08* |  | -0.53* | -0.68 | 0.25 |
| CL_L | | | | 0.23* | | 0.11 | 0.31 |  | 0.71* | | 0.22 | | 0.42 |  | 0.67* | -0.18 | 0.37 |
| CL_W | | | | -0.72* | | 0.43 | -0.06 |  | -0.58* | | -0.39 | | 0.16 |  | -0.60* | -0.61 | 0.13 |
| CA_L | | | | -0.65* | | -0.55 | 0.05 |  | -0.81* | | 0.03 | | -0.19 |  | -0.77* | 0.31 | 0.25 |
| CA_W | | | | -0.61* | | -0.43 | 0.14 |  | -0.77* | | -0.14 | | 0.21 |  | -0.56* | 0.29 | 0.22 |
|  |  |  |  | |  |  |  |  | |  | |  |  |  |  |  |  |

*Eigen scores >0.50. DS_L: Length of the dorsal sepal; DS_W: Width of the dorsal sepal; PT_L: Length of the petal; PT_W: Width of the petal; CO_L: Length of the column; CO_W: Width of the column; LL_L: Length of the lateral lobe of the lip; LL_W: Width of the lateral lobe of the lip; CL_L: Length of the central lope of the lip; CL_W: Width of the central lope of the lip; CA_L: Length of the callus; CA_W: Width of the callus.
