# Supplementary material for: Outcomes of Extensive Hybridization and Introgression in Epidendrum (Orchidaceae): Can We Rely on Species Boundaries?
Source: PLoS One. 2013 Nov 5;8(11):e80662. doi: 10.1371/journal.pone.0080662 (PMC3818259; doi:10.1371/journal.pone.0080662)
Supplement: Table S6 — Analysis of variation under a Univariate General Linear Model of four phenological parameters: onset, peak, termination and duration of flowering. *, **, *** indicates significant differences at P = 0.05, P = 0.01, and P = 0.001 respectively. (DOCX) [file pone.0080662.s008.docx]

**Table S6.** Analysis of variation under a Univariate General Linear Model of four phenological parameters: onset, peak, termination and duration of flowering. *, **, *** indicates significant differences at *P*=0.05, *P*=0.01, and *P*=0.001 respectively.

| **Parameter** | **Source** | **Type III SS** | **df** | **MS** | ***F*** | ***P*** |
| --- | --- | --- | --- | --- | --- | --- |
| **Onset** | Species | 8050.27 | 2 | 4025.13 | 3870.31 | 0.0001*** |
|  | Population | 650.64 | 8 | 81.33 | 77.90 | 0.002** |
|  | Species x Population | 91.72 | 4 | 22.93 | 22.04 | 0.001** |
|  | Error | 3.13 | 3 | 1.04 |  |  |
|  | Total | 8795.76 | 17 |  |  |  |
| **Peak** | Species | 40.35 | 2 | 20.17 | 288.14 | 0.0001*** |
|  | Population | 206.13 | 8 | 25.76 | 368.01 | 0.0001*** |
|  | Species x Population | 16.82 | 4 | 4.205 | 60.07 | 0.003** |
|  | Error | 0.21 | 3 | 0.07 |  |  |
|  | Total | 263.51 | 17 |  |  |  |
| **Termination** | Species | 5613.87 | 2 | 2806.93 | 10396.03 | 0.0001*** |
|  | Population | 479.68 | 8 | 59.96 | 222.07 | 0.0001*** |
|  | Species x Population | 38.36 | 4 | 9.59 | 35.51 | 0.007** |
|  | Error | 0.82 | 3 | 0.27 |  |  |
|  | Total | 6132.73 | 17 |  |  |  |
| **Duration** | Species | 8050.27 | 2 | 4025.13 | 3870.31 | 0.0001*** |
|  | Population | 650.64 | 8 | 81.33 | 78.2 | 0.002* |
|  | Species x Population | 91.72 | 4 | 22.93 | 22.04 | 0.01* |
|  | Error | 3.13 | 3 | 1.04 |  |  |
|  | Total | 8795.76 | 17 |  |  |  |
| **Overlap** | Species | 11519.3 | 2 | 5759.65 | 52360.45 | 0.0001*** |
|  | Population | 2308.12 | 8 | 461.62 | 4196.54 | 0.0001*** |
|  | Species x Population | 94.43 | 4 | 23.6 | 214.54 | 0.0001*** |
|  | Error | 1.37 | 3 | 0.11 |  |  |
|  | Total | 13923.22 | 17 |  |  |  |
